# Predicting skin graft failure on the scalp by intraoperative laser speckle analysis^☆^

**DOI:** 10.1016/j.abd.2023.05.009

**Published:** 2024-04-12

**Authors:** André Pinho, Ana Brinca, Carolina Figueiredo, Duarte Flor, Ricardo Vieira

**Affiliations:** a Dermatology Department, Coimbra Hospital and University Centre, Coimbra, Portugal; b Faculty of Medicine, University of Coimbra, Coimbra, Portugal

Dear Editor,

Reconstruction with skin grafts after cutaneous malignancy excision might carry some graft failure despite the best surgical practice.^1^, ^2^ It is well-established that a poorly perfused recipient bed has a higher risk of skin graft failure.^3^

The vascular supply for skin grafts on the scalp derives mainly from aponeurotic galea, but in a dermato-oncologic setting, its integrity is often compromised.^4^ By week four, the failure rate of full-thickness skin grafts (FTSG) on the scalp can reach 22%.^1^

The impact of the perfusion status of the recipient bed on graft viability at week four still needed to be quantified in humans *in vivo*.^3^

Laser speckle contrast imaging (LSCI) is a non-invasive, real-time, contactless technique that allows the study of microcirculation on large surface areas intra- and perioperatively.^5^ In previous research, we validated the study of microcirculation of distinct types of skin grafts by LSCI analysis.^6^ Its role in identifying high-risk patients for graft necrosis still needs to be explored.

In this pilot study, we aim to compare the outcomes of FTSG on the scalp with the baseline graft bed perfusion assessed by LSCI. We will also describe the changes in FTSG perfusion over four weeks.

After local ethics committee approval and informed consent, four consecutive patients with non-melanoma skin cancer of the scalp were enrolled: all male gender with 71y/o (patient 1), 90y/o (patient 2), 81y/o (patient 3) and 78y/o (patient 4). The main characteristics and comorbidities of the patients are presented in Table 1. The tumors were excised after lidocaine with epinephrine infiltration, and FTSG were harvested from the infraclavicular area. Twenty minutes after lesion removal and before skin graft suture, baseline wound bed perfusion (in arbitrary perfusion units, APU) was measured with LSCI for 1 minute, and mean arterial pressure (MAP, in mmHg) was registered. The ratio APU/MAP yielded cutaneous vascular conductance (CVC, in APU/mmHg), allowing the comparison of patients' perfusion values. FTSG was ultimately sutured, and tie-over-dressing was applied for one week.

**Table 1:** Characteristics and comorbidities of the (male) patients submitted to skin graft on the scalp.

Patient	Age	Excised tumour	Cardiovascular risk factors	Antiplatelets or anticoagulants	Other comorbidities
1	71	LM	AHT	Aspirin	Inguinal hernia
			Dyslipidemia		
2	90	LMS	AHT	None	Glaucoma
			Dyslipidemia		Osteoarthritis
3	81	SCC	AHT	None	Transplanted kidney
			Dyslipidemia		
4	78	BCC	AHT	None	CAD
			Dyslipidemia		
			T2DM		

AHT, Arterial Hypertension; BCC, Basal Cell Carcinoma; CAD, Coronary Arterial Disease; LM, Lentigo Maligna; LMS, Leiomyosarcoma; SCC, Squamous Cell Carcinoma; T2DM, Type 2 Diabetes Mellitus.

By day (D) 7, D14, D21, and D28, graft perfusion was assessed and compared with baseline perfusion of graft bed on D0. Clinical pictures were also registered. Necrosis areas were calculated with SketchAndCalc Area Calculator™ (version 6.2.5, 2018).

In all patients, skin cancer removal allowed the preservation of some underlying aponeurotic galea for skin graft vascular supply. No patient developed a postoperative infection, hematoma, or hemorrhage.

The primary defect areas were 8.4 cm^2^ (patient 1), 12.3 cm^2^ (patient 2), 5.8 cm^2^ (patient 3), and 8.7 cm^2^ (patient 4). The evolution of CVC values over the four weeks is represented in Fig. 1. The sequential clinical and LSCI images registered during the same period are presented in Fig. 2.

**Figure 1:**
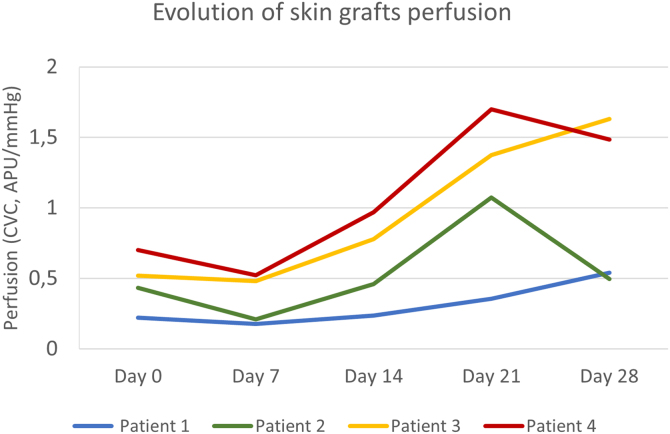
Evolution of full-thickness skin grafts perfusion on the scalp. Skin graft perfusion on day 7 was lower than baseline graft bed perfusion in all patients. Patients 3 and 4 had higher baseline perfusion and reached peak values on day 21 and day 28, respectively. Patient 1, with the worst clinical outcome, had an ascending curve until day 28 but with persistently lower perfusion values.

**Figure 2:**
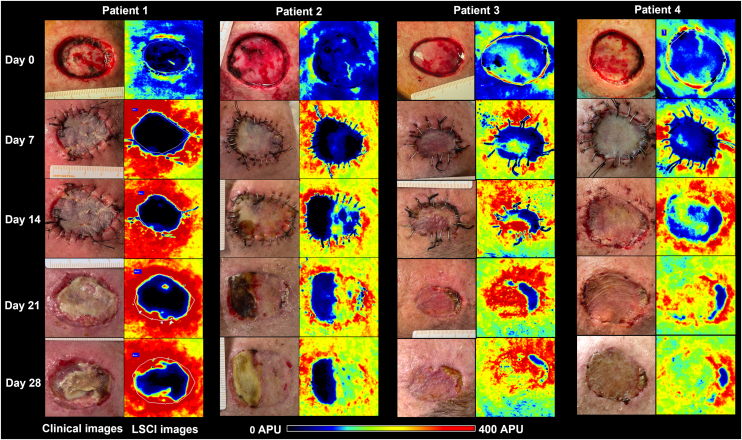
Clinical (left panels) and LSCI (right panels) images of full-thickness skin grafts on the scalp of the four patients. LSCI images show differences in perfusion in a semiquantitative scale, from black (meaning no perfusion), to red (intense perfusion). Patient 1 had a bluish-to-black recipient bed on day 0, and the graft remained poorly perfused until day 28, culminating in subtotal graft necrosis. Patient 2 had a bluish graft bed (meaning low perfusion) but gradually recovered over four weeks, and the graft necrosis area was less than 50%. Patients 3 and 4 had better-perfused graft beds and showed intense perfusion after day 14 onwards. By day 28, necrosis extensions were 16% and 9%, respectively. LSCI, laser speckle contrast Imaging; APU, arbitrary perfusion units.

Baseline CVC values of the recipient bed were 0.22 APU/mmHg, 0.43 APU/mmHg, 0.51 APU/mmHg, and 0.70 APU/mmHg, respectively, on patients 1, 2, 3, and 4. The graft necrosis extension on day 28 decreased with increased recipient bed perfusion (CVC) on day 0. It was 82% on patient 1, 48% on patient 2, 16% on patient 3, and 9% on patient 4.

After an initial decrease from day 0 to 7, by day 14, graft CVC overcame baseline recipient bed CVC in all patients. The increase in graft perfusion was more remarkable from day seven until day 21, with a posterior tendency to decrease (in patients 2 and 4) or a slight increase (in patients 1 and 3). The lower the area under the perfusion curves, the higher the graft necrosis extension on day 28 in all patients.

From the analysis of LSCI images, we observed that from day 14 onwards, the regions of skin graft poorly perfused (black to bluish) remained relatively stable in extension. These poorly perfused regions on day 14 overlapped with the regions of clinical necrosis on day 28.

There are a few ways of evaluating skin perfusion non-invasively, but the evidence supporting their use in skin graft vascularisation monitoring is lacking. Optical coherence tomography requires sequential assessments of less than 1 cm^2^ along the skin graft surface. Also, direct contact with the skin graft might be inappropriate in an aseptic intraoperative setting.^7^ Laser Doppler Imaging (LDI) allows non-contact assessment of cutaneous blood flow, with excellent spatial resolution but poor temporal resolution, especially when assessing extensive areas.^8^ LDI has a penetration depth of 0.5‒1.0 mm, more profound than the superficial dermal plexus that composes the cutaneous microcirculation.^9^ LSCI overcomes most of the limitations of these techniques. It allows the quick acquisition of high-resolution perfusion images of extensive areas of graft surface without contact.^10^ The short penetration of the laser radiation (0.3 mm) embraces capillaries, venules, and arterioles of the superficial papillary plexus, including the graft vasculature.^9^

Regardless of the individual variables not analyzed and the small sample size not allowing any statistical correlation estimation, we showed that the area of FTSG necrosis increased with decreasing baseline perfusion. The grafts with the worst outcomes, 82% and 48% of necrosis, had the lowest baseline perfusions, as observed in patients 1 and 2, respectively. Moreover, in patient 1, with subtotal graft necrosis, the perfusion curve slope was more downward than the others.

We demonstrate that the perfusion status of the graft bed can affect the outcome of FTSG, thus corroborating the established empirical knowledge. From our knowledge, this is the first report of using LSCI intraoperatively to identify patients with poorly perfused recipient graft beds who are, in theory, at increased risk of graft necrosis. This methodology must be expanded in larger samples and high-risk areas for skin graft necrosis other than the scalp, such as plantar or leg surfaces.^3^ With more robust data, identifying a perfusion (CVC) threshold might make the skin graft delay a better option than immediate skin grafting after tumor excision. We also speculate that the differences in CVC curves along the first graft healing might help to predict a higher risk of late necrosis.

LSCI can be promising in selecting the best candidates for surgical reconstruction with FTSG and in the intraoperative prediction of late necrosis.

## Financial support

None declared.

## Authors’ contributions

André Pinho: Conceptualization (equal), investigation (lead), formal analysis (equal), and writing-original draft (lead).

Ana Brinca: Conceptualization (equal) and writing-review & editing (lead).

Ana Figueiredo: Investigation (supporting).

Duarte Flor: Investigation (supporting), formal analysis (supporting).

Ricardo Vieira: Conceptualization (supporting) and writing-review & editing (supporting).

## Conflict of interest

None declared.
